# Enzymatic hydrolysis of steam-pretreated lignocellulosic materials with *Trichoderma atroviride *enzymes produced in-house

**DOI:** 10.1186/1754-6834-2-14

**Published:** 2009-07-06

**Authors:** Krisztina Kovacs, Stefano Macrelli, George Szakacs, Guido Zacchi

**Affiliations:** 1 Department of Chemical Engineering, Lund University, Lund, Sweden; 2 Department of Applied Biotechnology and Food Science, Budapest University of Technology and Economics, Budapest, Hungary

## Abstract

**Background:**

Improvement of the process of cellulase production and development of more efficient lignocellulose-degrading enzymes are necessary in order to reduce the cost of enzymes required in the biomass-to-bioethanol process.

**Results:**

Lignocellulolytic enzyme complexes were produced by the mutant *Trichoderma atroviride *TUB F-1663 on three different steam-pretreated lignocellulosic substrates, namely spruce, wheat straw and sugarcane bagasse. Filter paper activities of the enzymes produced on the three materials were very similar, while β-glucosidase and hemicellulase activities were more dependent on the nature of the substrate. Hydrolysis of the enzyme preparations investigated produced similar glucose yields. However, the enzymes produced in-house proved to degrade the xylan and the xylose oligomers less efficiently than a commercial mixture of cellulase and β-glucosidase. Furthermore, accumulation of xylose oligomers was observed when the TUB F-1663 supernatants were applied to xylan-containing substrates, probably due to the low β-xylosidase activity of the enzymes. The efficiency of the enzymes produced in-house was enhanced by supplementation with extra commercial β-glucosidase and β-xylosidase. When the hydrolytic capacities of various mixtures of a commercial cellulase and a *T. atroviride *supernatant produced in the lab were investigated at the same enzyme loading, the glucose yield appeared to be correlated with the β-glucosidase activity, while the xylose yield seemed to be correlated with the β-xylosidase level in the mixtures.

**Conclusion:**

Enzyme supernatants produced by the mutant *T. atroviride *TUB F-1663 on various pretreated lignocellulosic substrates have good filter paper activity values combined with high levels of β-glucosidase activities, leading to cellulose conversion in the enzymatic hydrolysis that is as efficient as with a commercial cellulase mixture. On the other hand, in order to achieve good xylan conversion, the supernatants produced by the mutant have to be supplemented with additional β-xylosidase activity.

## Background

Lignocellulosic materials, including agricultural residues, softwoods and hardwoods, are composed of cellulose, hemicellulose and lignin, and have great potential as cheap and renewable feedstocks for bioethanol production. The cellulose fraction of various lignocelluloses is a uniform structure consisting of β-1,4 linked glucose units. However, the biodegradability of cellulose may vary between plants, depending on the strength of association of the cellulose with other plant compounds. The composition and proportion of hemicellulose and lignin are highly dependent on the nature of the material. There is more lignin in softwoods (for example, spruce) than in hardwoods (for example, willow) or agricultural residues (for example, wheat straw or sugarcane bagasse), which makes softwood a particularly challenging material for ethanol production. The major hemicellulose component of hardwood and agricultural residues is xylan, while that of softwood is mostly mannan [1].

A great deal of research has focused on the enzymatic degradation of lignocellulosic polysaccharides into monomer sugars which can then be converted into fuel ethanol [2-5]. In an economically viable biomass-to-bioethanol process, utilization of both the cellulose and the hemicellulose part is desirable. The complete hydrolysis of cellulose and hemicellulose requires a well-designed cocktail of enzymes consisting of endoglucanases, cellobiohydrolases, β-glucosidases, xylanases, mannanases and various enzymes acting on side chains of xylans and mannans. Due to the recalcitrant structure of lignocelluloses, a pretreatment step is needed prior to enzymatic hydrolysis in order to make the cellulose more accessible to the enzymes [6]. In acid-catalyzed pretreatment, the major part of the hemicellulose is degraded, and the cellulose has to be hydrolyzed by the use of cellulases, while in alkali-catalyzed pretreatment, part of the lignin is removed, and in addition to cellulases, hemicellulases are also needed to hydrolyze the remaining polysaccharides [7].

The enzyme systems of *Trichoderma reesei *are the most extensively investigated. This species produces numerous cellulose- and hemicellulose-degrading enzymes. However, the extracellular β-glucosidase secretion is low, leading to incomplete hydrolysis of the cellulose [8-10]. It is therefore important to supplement *T. reesei *cellulases with extra β-glucosidase activity in order to obtain high cellulose conversion. Another option may be the use of enzymes produced by other, good β-glucosidase-producing fungi, for example, various *Penicillium *strains [11,12] or *T. atroviride *mutants [13,14]. Enzyme production is an important step in the biomass-to-ethanol process. Since the process is highly sensitive to the cost of the raw material, on-site enzyme production on part of the lignocellulose substrate already available in the ethanol plant would be advantageous [15].

The regulation of cellulase and hemicellulase production in *Trichoderma *has been studied in detail, and it has been proven that different degradation products formed during the hydrolysis of lignocellulose can induce the genes encoding for the different cellulases and hemicellulases [16]. It has also been hypothesized that cultivation of the fungus on a given lignocellulosic substrate would result in an enzyme profile especially suitable for the hydrolysis of that particular substrate. However, no clear relationship between the substrate used for cultivation and the hydrolytic performance of the resulting enzymes on the particular substrates has been reported in previous studies on *T. reesei *Rut C30 [17] or *Penicillium brasilianum *[11].

We recently reported good extracellular production of cellulase and β-glucosidase by *T. atroviride *mutants on steam-pretreated willow (SPW) [13] and steam-pretreated spruce (SPS) [14]. Using the *T. atroviride *enzymes produced, glucose yields as high as with commercial enzymes were achieved in the hydrolysis of SPW and SPS. However, degradation of the hemicellulose part of the substrates was not investigated.

In the present study, enzymes were produced on the whole steam-pretreated slurries of spruce, wheat straw (SPWS) and sugarcane bagasse (SPB) with the mutant *T. atroviride *TUB F-1663, and subsequent hydrolysis of the lignocellulosic substrates with the enzymes produced in-house was investigated. The hydrolytic potential, in terms of glucose and xylose yields obtained with these enzymes, was evaluated and compared with that of a commercial cellulase mixture.

## Methods

### Steam pretreatment

Spruce was kindly provided by a sawmill in southern Sweden (Widtsköfle Sågwerk AB, Degeberga, Sweden), wheat straw was obtained from a local riding school (Lund, Sweden), and sugarcane bagasse was kindly provided by Florida Crystals Corporation (Okeelanta, FL, USA). The lignocellulosic materials were steam pretreated at different conditions (see Table 1) in a steam-pretreatment unit equipped with a 10-L reactor vessel described by Stenberg *et al*. [18]. The pretreated slurries were thoroughly mixed and stored at 4°C until use. The total dry matter (DM) and water-insoluble solids (WIS) content of the SPS, SPWS and SPB were evaluated. Soluble sugar monomers, oligomers and degradation products were determined from the liquid fraction according to the standardized methods of the National Renewable Energy Laboratory (NREL, Golden, CO, USA) [19]. The solid residue was washed with water in order to remove the soluble substances, and the composition of the WIS was determined according to Sluiter *et al*. [20].

**Table 1:** Steam pretreatment conditions

Substrate	Spruce	Wheat straw	Bagasse
Size (mm)	2–10	2–10	Not determined

Prior to pretreatment	Impregnated with 2.5% SO_2 _(w/w moisture)	Soaked in 0.2% H_2_SO_4 _solution (20 g solution/g dry straw)	Impregnated with 2% SO_2 _(w/w moisture)

Pretreatment temperature (°C)	210	190	200

Pretreatment time (min)	5	10	5

### Commercial enzymes

Celluclast 1.5 L (Cell), a cellulase mixture produced by *T. reesei*, Novozym 188 (Nov), a β-glucosidase preparation from *Aspergillus niger*, and Pulpzyme HC (P), a xylanase enzyme produced by a genetically modified *Bacillus *species were kindly provided by Novozymes A/S (Bagsvaerd, Denmark). Multifect xylanase (M), a commercial *Trichoderma sp*. xylanase preparation was kindly provided by Genencor, Danisco A/S (Copenhagen, Denmark).

### In-house-produced enzymes

The mutant *T. atroviride *TUB F-1663 came from the Technical University of Budapest collection. Isolation and mutagenesis data for this strain have been described in a previous study [13]. The freeze-dried culture was revitalized and maintained on potato-dextrose-agar Petri plates at 30°C. Properly sporulating cultures were used for inoculation.

Enzyme production with *T. atroviride *TUB F-1663 was performed on SPS, SPWS and SPB in 2-L Labfors fermentors (Infors AG, Bottmingen, Switzerland) with a working volume of 1 L. The composition of the media was the following: (in g/L): SPS, SPWS or SPB (whole slurries), 15 (total DM); KH_2_PO_4_, 1.5; (NH_4_)_2_HPO_4_, 2; soybean meal, defatted, 1; corn steep liquor (50% DM, Sigma), 1; NaCl, 0.5; MgSO_4·_7H_2_O, 0.3; urea, 0.3; CaCl_2_, 0.3; and CaCO_3_, 1, and (in mg/L): CoCl_2_·6H_2_O, 2; MnSO_4_, 1.6; ZnSO_4_·7H_2_O, 3.45; and FeSO_4_·7H_2_O, 5. The pH before inoculation was 5.2.

Fermentors containing 900 ml of the medium were sterilized in an autoclave (*ex situ*) at 121°C for a minimum of 30 min. Spores were harvested aseptically using a loop from one Petri plate culture and suspended in 100 ml sterile water. The spore suspensions were added to the fermentors after sterilization and cooling. The experiments were performed in duplicate at 30°C, and the pH was controlled in the range of 4.5–5.6 by adding 5% NaOH and 5% H_2_SO_4_. The fermentors were aerated at a flow rate of 0.3 L/min, corresponding to 0.3 (v/v)/min. The dissolved oxygen level was maintained above 25% by varying the agitation speed (350–650 rpm). An antifoam agent (Dow Corning Antifoam RD Emulsion, VWR International Ltd.) was added manually (1–2 drops) when foaming was observed. Enzyme production was continued for 96 h, and samples were withdrawn roughly every 24 h. Filter paper activity (FPA) and β-glucosidase activity were measured in each sample. After fermentation the whole broths were centrifuged at 4,000 rpm for 10 min, the supernatants were filtered through a 0.2 μm nylon filter (Pall Corporation, New York, USA), and the enzyme filtrates were maintained at 4°C until further use.

### Enzyme assays

Total reducing sugars were determined colorimetrically using the dinitrosalicylic acid method [21]. Filter paper activity (FPA) and endoglucanase activity were measured as described by Ghose [22] with Whatman No. 1 filter paper and 1% (w/v) hydroxyethyl cellulose (Fluka) as substrates. The xylanase activity was determined according to Bailey *et al*. [23] using 1% (w/v) birchwood xylan (Sigma) as substrate. The mannanase activity was measured as described by Stalbrand *et al*. [24] with 0.5% (w/v) locust bean gum (Sigma) as substrate. β-glucosidase activity was assayed with p-nitrophenyl-β-D-glucopyranoside (PNPG, Sigma) using Berghem and Pettersson's method [25] with slight modifications. The assay mixture contained 1 ml 5 mM PNPG in 0.05 M sodium acetate buffer (pH 4.8) and 100 μl appropriately diluted enzyme solution. After incubation at 50°C for 10 min, 2 ml 1 M Na_2_CO_3 _was added to the mixture. After cooling, the sample was diluted with 10 ml distilled water and the liberated p-nitrophenol (PNP) was measured at 400 nm. One international unit of β-glucosidase activity (IU) liberates 1 μmol PNP per min under the assay conditions. The β-xylosidase and the β-mannosidase activities were measured with 5 mM p-nitrophenyl-β-D-xylopyranoside (Sigma) and 5 mM p-nitrophenyl-β-D-mannopyranoside (Sigma), respectively, analogously to the β-glucosidase assay.

### Enzymatic hydrolysis

For simplicity, the enzyme supernatants of *T. atroviride *TUB F-1663 produced on SPS, SPWS and SPB are henceforth referred to as F-1663/S, F-1663/WS and F-1663/B, respectively, while the 3:1 mixture of Celluclast 1.5 L and Novozym 188 is denoted Cell+Nov. In order to investigate the influence of the substrate used for enzyme production on hydrolysis, the F-1663/S, F-1663/WS and F-1663/B enzyme supernatants and the Cell+Nov mixture were used in duplicate to hydrolyze the three substrates at an enzyme loading of 3 FPU (filter paper unit)/g WIS.

The effect of the addition of extra β-glucosidase and β-xylosidase enzymes to the produced F-1663/B supernatant was studied in the hydrolysis of SPB. Celluclast 1.5 L (Cell) was used for comparison in this experiment. The enzyme loadings of F-1663/B and Cell were 3 FPU/g WIS, that of Novozym 188 (Nov) 7.6 β-glucosidase IU/g WIS, while Multifect xylanase (M) and Pulpzyme (P) were applied at 2.7 and 5.9 β-xylosidase IU/g WIS, respectively. The experiment was performed in duplicate.

When studying the hydrolytic capacities of various Cell & F-1663/B mixtures on SPB, the enzymes were mixed together at FPA ratios of 1:0; 2:1; 1:1; 2:5 and 0:1. The resulting FPA of each mixture was measured and hydrolysis was performed at an enzyme loading of 3 FPU/g WIS.

The unwashed pretreated substrates (whole slurries) were diluted with 0.1 M sodium acetate buffer (pH 4.8) to 20 g/L WIS in a total volume of 250 ml. Hydrolysis was carried out at 40°C for 96 h. Samples were centrifuged at 3,000 rpm for 5 min and filtered through 0.2 μm filters (MFS-13, Micro Filtration Systems, Dublin, CA, USA) before analysis.

### Analytical methods

The composition of the pretreated materials and samples following enzymatic hydrolysis was analyzed using a high performance liquid chromatography instrument (Shimadzu Kyoto, Japan) equipped with a refractive index detector (Shimadzu). Cellobiose, glucose, xylose, galactose, arabinose and mannose were separated using an Aminex HPX-87P column (Bio-Rad, Hercules, CA, USA) at 80°C, with deionized water as eluent at a flow rate of 0.5 ml/min, while lactic acid, glycerol, acetic acid, hydroxymethylfurfural and furfural were separated on an Aminex HPX-87H column (Bio-Rad) at 65°C, with 5 mM H_2_SO_4 _as eluent at a flow rate of 0.5 ml/min.

## Results and discussion

### Steam-pretreated substrates

The composition of the steam-pretreated materials is given in Table 2. The major insoluble compound was glucan (approximately 55–65% of WIS) in all substrates; however, significant differences in the composition of other components were seen. The solid fraction of SPS contained 42.3% lignin and 2.3% mannan, that of SPWS contained 27.2% lignin and 2.1% xylan, while that of SPB had 27% lignin and 6.7% xylan. The main soluble compounds proved to be glucose, mannose and xylose in SPS, while the liquid fraction of SPWS and SPB contained mainly xylose and xylose oligomers.

**Table 2:** Composition of the steam-pretreated substrates

		Spruce (SPS)		Wheat straw (SPWS)		Bagasse (SPB)	
		g/L whole slurry	% of WIS	g/L whole slurry	% of WIS	g/L whole slurry	% of WIS

	Total DM	203.2	-	113.1	-	138.9	-
	WIS	135.2	-	77.1	-	98.5	-

Solid fraction	Glucan	73.1	54.0	50.2	65.1	59.6	60.5
	Xylan	n.d.	n.d.	1.6	2.1	6.6	6.7
	Galactan	0.7	0.5	0.5	0.6	n.d.	n.d.
	Arabinan	n.d.	n.d.	0.9	1.2	0.3	0.3
	Mannan	3.1	2.3	n.d.	n.d.	n.d.	n.d.
	Insoluble lignin	57.2	42.3	20.9	27.2	26.6	27.0

Liquid fraction	Glucose	22.4	-	2.3	-	2.6	-
	Glucose oligomers	1.7	-	0.6	-	1.3	-
	Xylose	9.7	-	21.8	-	10.9	-
	Xylose oligomers	n.d.	-	3.5	-	18.1	-
	Galactose	4.1	-	1.1	-	0.8	-
	Galactose						
	oligomers	n.d.	-	0.1	-	0.7	-
	Arabinose	2.3	-	3.3	-	1.5	-
	Arabinose						
	Oligomers	n.d.	-	n.d.	-	0.6	-
	Mannose	20.9	-	n.d.	-	0.8	-
	Mannose						
	oligomers	0.1	-	n.d.	-	n.d.	-
	Lactic acid*	2.4	-	0.1	-	0.5	-
	Glycerol	n.d.	-	n.d.	-	0.3	-
	Acetic acid	4.6	-	2.1	-	3.6	-
	HMF	2.3	-	0.3	-	0.1	-
	Furfural	1.4	-	1.0	-	1.0	-
	Soluble lignin	2.3	-	2.1	-	3.0	-

WIS, water-insoluble solids; DM, dry matter; HMF, hydroxymethylfurfural, n.d., not detected

*The lactic acid component was probably some other organic acid that had the same retention time as lactic acid.

The values presented are the average of two separate measurements, and the standard deviations are <5%.

### Enzyme production

The mutant *T. atroviride *TUB F-1663 was cultivated on three different pretreated lignocellulosic materials (SPS, SPWS and SPB) in order to produce cellulolytic enzymes for hydrolysis. The concentration of the substrates was 15 g/L DM (whole slurry), which corresponded to 10.0, 10.2 and 10.6 g/L WIS for SPS, SPWS and SPB, respectively. The measured activities of the enzymes produced after 96 h of fermentation and also those of the commercial enzymes are summarized in Table 3.

**Table 3:** Activities of the enzymes used in hydrolysis

Enzyme				Enzyme activity			
	FPA (FPU/g)	β-glucosidase (IU/g)	Xylanase (IU/g)	β-xylosidase (IU/g)	Endoglucanase (IU/g)	Mannanase (IU/g)	β-mannosidase (IU/g)
Celluclast 1.5 L	56.2	30.8	750	63.6	13087	122	~0.08
Novozym 188	n.d.	502	34.7	6.7	296	17.9	5.4
Multifect xylanase	0.15	26.6	22969	26.5	454	n.a.	n.a.
Pulpzyme HC	17.9	12.9	1343	118.2	6961	n.a.	n.a.
F-1663/S	0.36	7.6	5.0	<0.01	71.6	1.1	~0.02
F-1663/WS	0.36	5.2	39.5	~0.05	72.7	0.98	~0.02
F-1663/B	0.37	4.8	40.1	~0.03	69.6	0.88	~0.02

FPA: filter paper activity; n.d.: not detectable.; n.a.: not analyzed.

F-1663/S, WS, B: the crude enzyme supernatants of *Trichoderma atroviride *TUB F-1663 produced on steam-pretreated spruce, wheat straw and sugarcane bagasse.

The activities presented are the mean values of at least two separate measurements. The standard deviations are <10%, and mainly 1–4% for FPA, <10% for β-glucosidase, 1–5% for xylanase and β-xylosidase, mostly around 10% for endoglucanase, and 5–10% for mannanase and β-mannosidase measurement.

There were no significant differences in the final FPA values (0.36–0.37 FPU/g) or endoglucanase activities (70–73 IU/g) obtained with the mutant TUB F-1663 on the three substrates, which suggested that cellulase production was only affected by the quantity and not the quality of the carbon source. On the other hand, there was a clear positive correlation between xylanase activity and the xylan content of the substrates; higher xylanase activities being measured on SPWS and SPB (approximately 40 IU/g) than on SPS (5 IU/g).

β-xylosidase is a key enzyme in the hydrolysis of xylan, that is, a high ratio of β-xylosidase to xylanase should indicate a good capacity to hydrolyze the xylan backbone. In the case of the F-1663 enzymes, the (β-xylosidase/xylanase)*100 ratio was found to be around 0.1–0.2, which was considerably lower than that measured in Cell (8.5), or previously reported for *T. reesei *culture filtrates (9.0), [26]. Despite the relatively low β-xylosidase activities of the three culture filtrates, it is clear that somewhat more β-xylosidase was produced on SPWS and SPB, which contained xylan and xylan-degradation products, than on SPS. This is in accordance with earlier studies, in which it was reported that the production of xylan-degrading enzymes was induced by the presence of xylan in the cultivation medium [11,17].

The mutant TUB F-1663 proved to be a good extracellular β-glucosidase-producing strain in previous studies on SPW [13] and SPS [14]. In the present study, high β-glucosidase activities were also measured on SPWS (5.2 IU/g) and SPB (4.8 IU/g), but the highest activity was detected on SPS (7.6 IU/g). For comparison, *T. reesei *Rut C30, a well-known hypercellulolytic strain, produced only around 0.1 IU/g β-glucosidase on SPS [14] and SPWS (data not shown).

### Enzymatic hydrolysis

The first aim of this study was to investigate the relationship between the cultivation substrates and the hydrolytic efficiency of the resulting enzyme preparations towards the particular substrates. The enzymes produced in-house on SPS, SPWS and SPB were used at equal loadings (FPA per g WIS) to hydrolyze the three pretreated materials (whole slurries). The results were compared with those obtained using the commercial enzyme mixture Cell+Nov.

Table 4 gives the final concentrations and yields of glucose and xylose achieved in the hydrolysis of SPS, SPWS and SPB. Results for mannose, arabinose and galactose are not presented, since their levels were almost constant during hydrolysis. The theoretical final glucose concentrations were similar for SPS, SPWS and SPB (15.6, 15.5 and 14.5 g/L, respectively); however, the glucose concentrations obtained after hydrolysis were significantly different. The highest levels were achieved on SPWS (9.6–10.4 g/L), while the lowest levels were obtained on SPS (6.8–6.9 g/L). The glucose yields, based on the hydrolysis of the glucan part of the substrates, were also considerably lower on SPS (approximately 29%) than on SPWS (60–65%) or SPB (50–55%). This was probably due, in part, to the rigid structure and the high lignin content of SPS [6]. It is known that the presence of lignin impedes the performance of the enzyme during hydrolysis by creating non-productive enzyme-lignin bonds [27,28]. It has been reported previously that partial delignification of pretreated materials before enzymatic hydrolysis results in increased sugar yields [29,30]. Furthermore, lower conversion of cellulose in SPS may also be related to the fact that the SPS slurry contained a much higher amount of glucose (around 3.3 g/L) than SPWS (approximately 0.9 g/L) and SPB (approximately 0.8 g/L) at the start of hydrolysis, which could have had a negative effect on the rate of hydrolysis [3,31-33]. Another reason for the lower glucose yields obtained on SPS may be that the spruce was pretreated at higher severity than wheat straw and sugarcane bagasse (Table 1), which could have resulted in higher concentrations of various degradation products that inhibited hydrolysis.

**Table 4:** Final concentrations and yields of glucose and xylose obtained in the hydrolysis of SPS, SPWS and SPB

Substrate	Enzyme	Final glucose (g/L)	Final xylose (g/L)	Glucose from hydrolysis (g/L)	Xylose from hydrolysis (g/L)	Glucose yield (% of theoretical^a^)	Xylose yield (% of theoretical^a^)
SPS	Cell+Nov	6.9	1.4	3.6	n.d.	29	-
	F-1663/S	6.9	1.4	3.6	n.d.	29	-
	F-1663/WS	6.8	1.4	3.5	n.d.	28	-
	F-1663/B	6.9	1.4	3.6	n.d.	29	-

SPWS	Cell+Nov	9.6	6.5	8.7	1.1	60	72
	F-1663/S	10.4	5.6	9.5	0.2	65	13
	F-1663/WS	10.3	5.6	9.4	0.2	64	13
	F-1663/B	10.0	5.6	9.1	0.2	62	13

SPB	Cell+Nov	7.6	5.6	6.8	3.0	50	53
	F-1663/S	7.6	3.0	6.8	0.4	50	7
	F-1663/WS	8.3	3.1	7.5	0.5	55	9
	F-1663/B	8.0	3.3	7.2	0.7	52	12

SPS: steam-pretreated spruce; SPWS: steam-pretreated wheat straw; SPB: steam-pretreated sugarcane bagasse

^a ^Theoretical sugar concentrations from hydrolysis (glucose and xylose originating from glucan + glucose oligomers and xylan + xylose oligomers, respectively) were 12.3 g/L glucose on SPS, 14.6 g/L glucose and 1.5 g/L xylose on SPWS, and 13.7 g/L glucose and 5.7 g/L xylose on SPB.

n.d.: not detected, Cell+Nov: the 3:1 (w:w) mixture of Celluclast 1.5 L & Novozym 188, F-1663/S, WS, B: the crude enzyme supernatants of *Trichoderma atroviride *TUB F-1663 produced on steam-pretreated spruce, wheat straw and sugarcane bagasse.

The values presented are the average of two separate hydrolysis experiments, and the standard deviations are <5%.

It can be seen from Table 4 that all the enzymes investigated performed similarly on SPS, in that no significant differences were observed in the glucose yields or in the sum of glucose and xylose concentrations. This latter finding is important in those processes where the microorganism used for ethanol production is also capable of fermenting pentoses [34]. The SPS slurry contained around 1.4 g/L xylose at the start of hydrolysis, and the concentration remained constant due to a lack of xylan or xylose oligomers in the substrate. SPS fibers contained 2.3% mannan (Table 2); however, no degradation of mannan was observed, that is, the concentration of mannose was stable during hydrolysis (data not shown), possibly due, in part, to the low β-mannosidase activity of the enzymes (Table 3).

**Figure 1:**
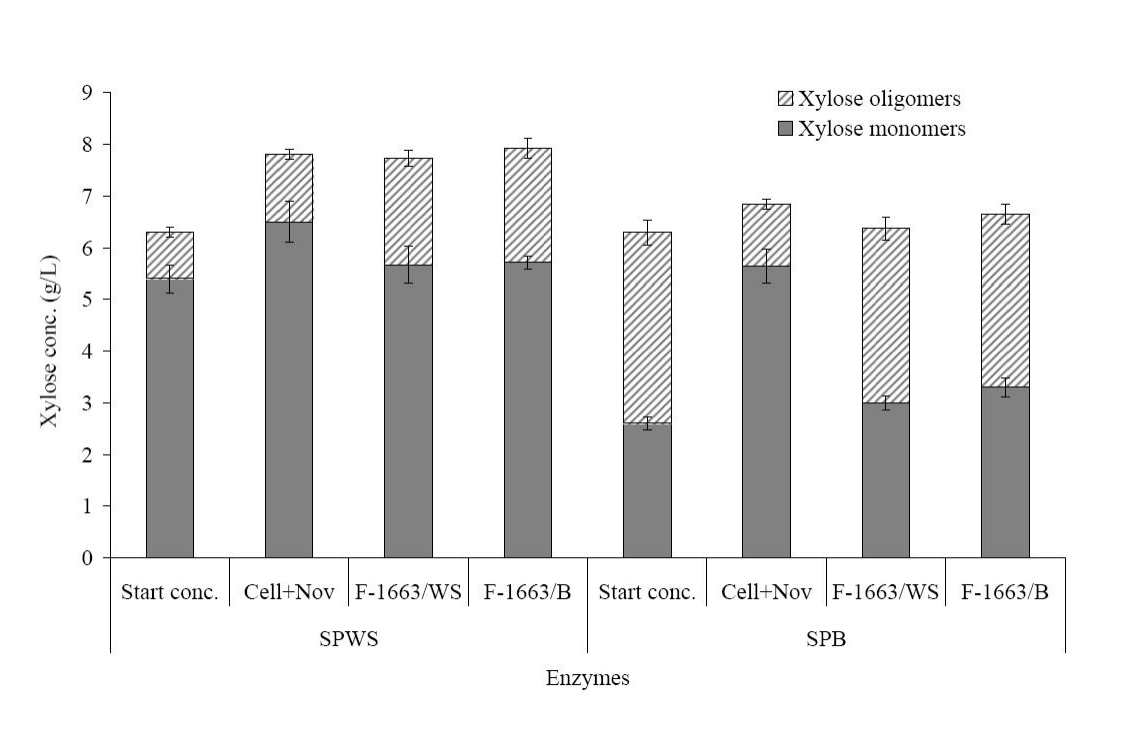
**Concentration of xylose monomers and oligomers at the start and after 96 h of hydrolysis**. Enzymatic hydrolysis of pretreated wheat straw (SPWS) and bagasse (SPB) (whole slurry) using the 3:1 (w:w) mixture of Celluclast 1.5 L & Novozym 188 (Cell+Nov), and the crude enzyme supernatants produced by *Trichoderma atroviride* TUB F-1663 on wheat straw (F-1663/WS) or bagasse (F-1663/B). The values presented are the average of two separate measurements. T: 40°C, pH: 4.8, substrate concentration: 20 g/L WIS, enzyme loading: 3 FPU/g WIS.

The sums of glucose and xylose monomers obtained with the different enzyme preparations on SPWS were very similar (15.6–16.1 g/L). In contrast, greater differences were seen on SPB, where a higher glucose + xylose concentration (13.2 g/L) was achieved with the commercial mixture than with the enzymes produced in-house (10.6–11.4 g/L glucose + xylose). The performance of the various enzymes differed mostly in their ability to degrade xylan and xylose oligomers. Xylose concentrations were found to be considerably lower in SPWS and SPB when the enzymes produced in-house were used than when Cell+Nov was employed (Table 4). In addition, higher levels of xylose oligomers were found in the hydrolysis slurry in the case of the F-1663 enzymes (Figure 1). The reason for this may be that although the F-1663 enzymes showed good xylanase activity, in contrast to Cell+Nov, their level of β-xylosidase was insufficient for the efficient degradation of xylan to xylose (Table 3). This is in accordance with a previous study on the hydrolysis of xylan using *T. reesei *culture filtrates in which it was reported that the xylose yield correlated better with the level of β-xylosidase than with that of xylanase [35]. In another study, both the glucose and the xylose yields achieved with in-house-produced *Penicillium brasilianum *enzymes were found to be higher than those obtained with a commercial Celluclast & Novozym mixture on wet-oxidized wheat straw [36]. This was probably due to the fact that, in contrast to the F-1663 enzymes, the *Penicillium brasilianum *enzymes had both higher β-glucosidase-to-FPA and higher β-xylosidase-to-FPA ratios than the commercial mixture.

Despite the good β-glucosidase activity of the F-1663 cellulases, surprisingly high amounts of cellobiose remained unhydrolyzed in SPWS and SPB (1.3–1.7 g/L) in comparison to SPS (approximately 0.5 g/L) (data not shown), suggesting that the effectiveness of a β-glucosidase could vary with the type of substrate, as reported earlier [37]. To investigate whether the accumulation of cellobiose could be reduced and the xylose yield increased when the F-1663/B supernatant is used in the hydrolysis of SPB, extra β-glucosidase (Nov) and β-xylosidase-containing xylanase enzymes (M and P) were added to the enzymes produced.

**Figure 2:**
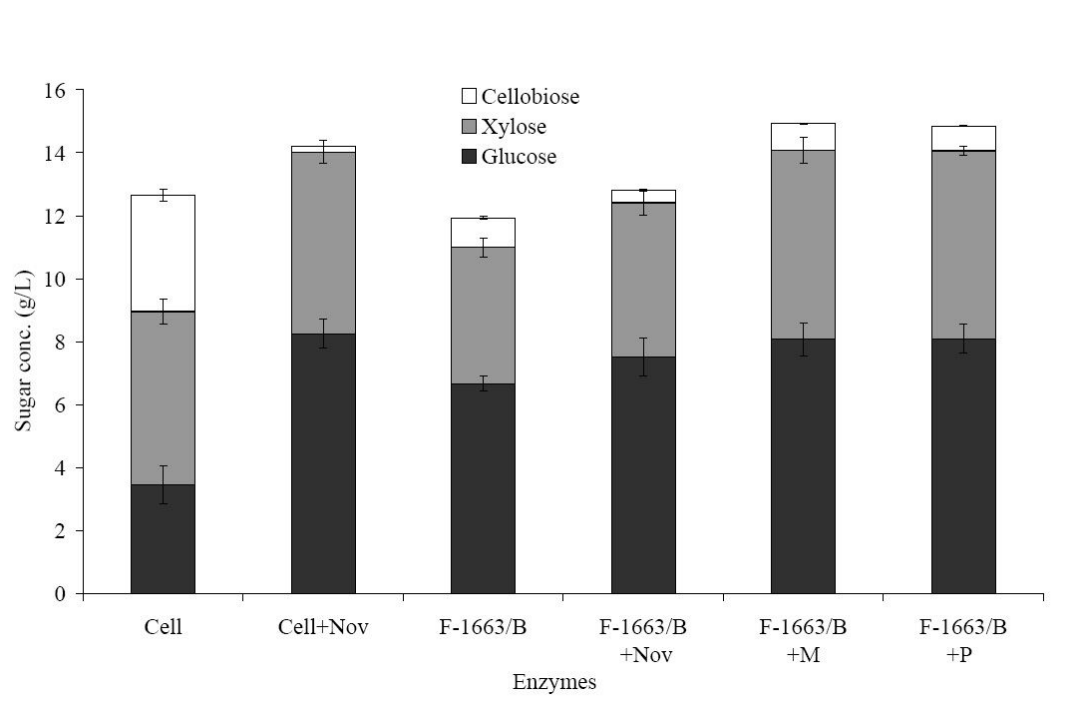
**Glucose, xylose and cellobiose concentrations after 96 h of hydrolysis of steam-pretreated sugarcane bagasse with various enzyme mixtures**. Cell: Celluclast 1.5 L (3 FPU/g WIS); Nov: Novozym 188 (7.6 β-glucosidase IU/g WIS); F-1663/B: enzyme supernatant of *Trichoderma atroviride* TUB F-1663 produced on pretreated bagasse (3 FPU/g WIS); M: Multifect xylanase (2.7 β-xylosidase IU/g WIS); and P: Pulpzyme (5.9 β-xylosidase IU/g WIS). The values presented are the average of two separate hydrolysis experiments. T: 40°C, pH: 4.8, substrate concentration: 20 g/L WIS.

Table 5 shows the measured specific β-glucosidase and β-xylosidase activities of the single enzymes Cell and F-1663/B, and the resulting specific β-glucosidase and β-xylosidase activities of the mixtures calculated based on the mixing ratio of the enzymes. F-1663/B was compared with Cell at the same FPA (3 FPU/g WIS). Due to higher specific β-glucosidase and lower specific β-xylosidase activities of F-1663/B, the final glucose concentration was found to be much higher, whereas that of xylose was lower with the enzymes produced than with Cell (Figure 2). The sum of glucose and xylose monomers obtained was 11 g/L with F-1663/B and 9 g/L with Cell. The addition of Nov to Cell considerably reduced the accumulation of cellobiose, from 3.7 g/L to 0.2 g/L, and subsequently increased the glucose concentration from 3.5 to 8.3 g/L. Although F-1663/B contained good β-glucosidase activity, supplementation of the enzyme with extra Nov was found to decrease the final cellobiose accumulation from around 1 g/L to 0.4 g/L, and improve the glucose level from 6.7 to 7.5 g/L. Similar results were obtained in a previous study, where improved saccharification was observed when extra Nov was added to *Penicillium *supernatants that already contained good β-glucosidase levels [12]. This could be explained by the fact that in addition to the high β-glucosidase activity, Nov also contains other activities (for example, endoglucanase, xylanase, β-xylosidase) (Table 3), which may contribute to the increased saccharification of the supplemented enzymes.

**Table 5:** Specific β-glucosidase and β-xylosidase activities (per g WIS) of the enzymes used in the hydrolysis of SPB

Enzyme	β-glucosidase (IU/g WIS)	β-xylosidase (IU/g WIS)
Cell	1.6	3.4
Cell+Nov	9.3	3.5
F-1663/B	39	0.24
F-1663/B+Nov	47	0.35
F-1663/B+M	42	2.9
F-1663/B+P	40	6.2

SPB: steam-pretreated sugarcane bagasse; WIS: water-insoluble solids

Cell: Celluclast 1.5 L (3 FPU/g WIS), Nov: Novozym 188 (7.6 β-glucosidase IU/g WIS).

F-1663/B: enzyme supernatant of *Trichoderma atroviride *TUB F-1663 produced on SPB (3 FPU/g WIS), M: Multifect xylanase (2.7 β-xylosidase IU/g WIS), P: Pulpzyme HC (5.9 β-xylosidase IU/g WIS).

The activities presented are calculated based on Table 3 and the mixing ratio of the enzymes.

Interestingly, although Cell supplemented with Nov had a lower specific β-glucosidase activity than the F-1663/B supplemented with Nov (Table 5), a higher final glucose concentration was obtained with Cell+Nov (8.3 g/L) than with F-1663/B+Nov (7.5 g/L) (Fig. 2). The reason for this could be the fact that the addition of Nov to Cell significantly increased the overall FPA of the resulting mixture [38]. In addition, Cell+Nov had a higher β-xylosidase content than F-1663/B+Nov (Table 5). An increase in xylose levels was also observed for both Cell (5.5%) and F-1663/B (14%) when additional β-glucosidase was used, which may have resulted from xylanase and β-xylosidase activities in Nov. Improvement of xylose yields by using Novozym 188 has also been reported by other authors [37].

Supplementation of F-1663/B with xylanase enzymes (M and P) resulted in an increase of 40% in the xylose level and an improvement of 21% in the glucose concentration. Previous studies have also shown that the addition of xylanase to cellulase enzymes enhanced both the glucan and the xylan conversions of a high-xylan-containing pretreated substrate, due to better accessibility of cellulose after xylan degradation [9,30]. Furthermore, it was suggested that supplementation of β-xylosidase improved glucose release during hydrolysis by decreasing the accumulation of xylose oligomers, which were found to inhibit cellulase activity [37].

**Figure 3:**
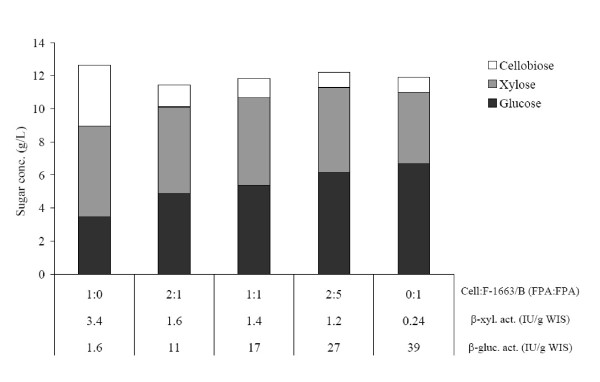
**Glucose, xylose and cellobiose concentrations after 96 h of hydrolysis of steam-pretreated sugarcane bagasse with various mixtures of a commercial cellulase and an enzyme produced in-house**. Cell: Celluclast 1.5 L, F-1663/B: enzyme supernatant of *Trichoderma atroviride* TUB F-1663 produced on pretreated bagasse. T: 40°C, pH: 4.8, substrate concentration: 20 g/L WIS, enzyme loading: 3 FPU/g WIS. See standard deviations for the single enzymes Cell and F-1663 in Figure 2.

The second aim was to investigate whether mixing of a good β-xylosidase- but poor β-glucosidase-containing enzyme (Cell) with a supernatant that has good β-glucosidase but low β-xylosidase activity (F-1663/B) would result in improved saccharification compared with the separate enzymes. Figure 3 shows that at the same enzyme loading (FPA) the final glucose and xylose concentrations obtained on SPB with various Cell & F-1663/B mixtures were correlated to the β-glucosidase and β-xylosidase activities of the mixtures, respectively. The lowest glucose (3.5 g/L) and highest xylose (5.5 g/L) concentrations were obtained with Cell, whereas the highest glucose (6.7 g/L) and lowest xylose (4.3 g/L) levels were achieved with F-1663/B. The 2:5 Cell:F-1663/B mixture proved to be slightly more efficient than the other enzyme mixtures tested in terms of the sum of glucose and xylose monomers (11.3 g/L). The sums of glucose and cellobiose concentrations were almost the same for Cell and F-1663/B due to the use of the same loading, but the percentage of glucose was much higher in the case of the high-β-glucosidase-containing F-1663/B enzyme (88%) than with the commercial enzyme (48%).

## Conclusion

Our previous studies have shown that when a high glucose concentration was desirable in the hydrolysis of pretreated lignocellulosic materials, enzymes derived from the mutant *T. atroviride *TUB F-1663 were competitive with commercial enzymes [13,14]. However, the present study showed that the supernatants of this mutant yielded significantly lower levels of xylose than a commercial enzyme mixture when pretreated substrates containing high levels of xylan and xylose oligomers were used. This could be explained by the relatively low ratio of β-xylosidase to xylanase activity in the F-1663 enzymes, resulting in the accumulation of xylose oligomers. *T. reesei *wild-type strains and mutants produce low levels of extracellular β-glucosidase while our *T. atroviride *mutant secretes suboptimal quantities of β-xylosidase.

In order to degrade all the polysaccharides in substrates containing a high amount of xylan and/or xylose oligomers to monomeric sugars for bioethanol production, supplementation of the enzymes produced by the mutant TUB F-1663 with additional β-xylosidase activity appears to be necessary in order to achieve good xylan conversion. Alternatively, more severe pretreatment conditions could be employed to break down the xylan content of the substrate more efficiently, rendering the addition of β-xylosidase in enzymatic hydrolysis unnecessary. However, at more severe pretreatment conditions, the concentration of the inhibitory compounds that limit the enzymatic hydrolysis would be higher. Another option could be the application of xylose oligomers accumulated during enzymatic hydrolysis for other purposes, such as biogas production [39].

In the future, screening for microorganisms capable of co-producing cellulases and xylanases with sufficient levels of both β-glucosidase and β-xylosidase enzymes may be of interest in the biomass-to-bioethanol process. The construction of recombinants by cloning and amplification of the genes encoding the enzymes produced at suboptimal quantities may also be an option.

## Competing interests

The authors declare that they have no competing interests.

## Authors' contributions

KK carried out the experiments, analyzed the results and wrote the manuscript. SM pretreated the bagasse and analyzed the composition of the pretreated bagasse before and after enzymatic hydrolysis. GS and GZ participated in the coordination of the study and reviewed the manuscript. All authors have read and approved the final manuscript.
